# Gain-Framed Messages Were Related to Higher Motivation Scores for Sugar-Sweetened Beverage Parenting Practices than Loss-Framed Messages

**DOI:** 10.3390/nu10050625

**Published:** 2018-05-16

**Authors:** Arwa Zahid, Marla Reicks

**Affiliations:** Department of Food Science and Nutrition, University of Minnesota, St. Paul, MN 55108, USA; zahid001@umn.edu

**Keywords:** parenting practices, sugar-sweetened beverages, gain- and loss-framed messages

## Abstract

Parents play an important role in promoting healthy beverage intake among children. Message-framing approaches, where outcomes are described as positive (gain) or negative (loss) results, can be used to encourage parenting practices that promote healthy beverage intakes. This study tested the effectiveness of message framing on motivation for parenting practices targeting reductions in child sugar-sweetened beverage (SSB) intake (controlling availability, role modeling) and dispositional factors moderating effectiveness. Parents (*n* = 380) completed a survey to assess motivation after viewing gain- and loss-framed messages to engage in parenting practices, usual beverage intake, and home beverage availability. Paired *t*-tests were used to examine differences in motivation scores after viewing gain- vs. loss-framed messages for all parents and by subgroups according to low vs. high SSB intake and home availability, and weight status. Gain- versus loss-framed messages were related to higher motivation scores for both parenting practices for all parents (*n* = 380, *p* < 0.01) and most subgroups. No differences were observed by message frame for parents in low home SSB availability or normal and overweight BMI subgroups for controlling availability. Gain- versus loss-framed messages were related to higher motivation scores, therefore gain-framed messages are recommended for parent interventions intended to decrease child intake of SSBs.

## 1. Introduction

Consumption of sugar-sweetened beverages (SSBs) by children in the United States (U.S.) is a concern because of high intake [1,2] and associated health problems [3,4,5,6]. National consumption data (2011–2014) showed that 62.9% youth aged 2–19 years in the U.S. consumed at least one SSB on a given day [2]. A recent review found consistent evidence for negative effects of SSB consumption on the health of children and adolescents, with strongest evidence for risk of overweight or obesity and dental caries [6]. Parenting practices, such as role modeling and controlling beverage availability are considered part of the social and physical environment which can be manipulated by parents to change SSB intake behaviors of children under the organizing framework of Social Cognitive Theory [7,8].

Message framing is a common method used to promote health behavior change [9,10]. Gain-framed messages (where outcomes are described in positive terms) have been found to be more effective for prevention behaviors (sunscreen use and exercise) whereas, loss-framed messages (where outcomes are described in negative terms) were more effective for detection behaviors (breast self-exams) [11,12]. In addition, a recent review suggested that gain-framed messages were more effective when the individual had a low level of involvement or interest in the issue, the outcome of the behavior was certain, and the behavior helped individuals avoid risk [10]. Another review found additional dispositional factors that consistently moderated motivation based on gain and loss framing of health messages including self-efficacy beliefs and ambivalence [13]. The frequency of SSB parenting practices, such as role modeling intake and controlling beverage availability, and weight status may reflect dispositional factors such as self-efficacy beliefs, level of involvement, or risk aversion. The limited number of studies that tested message framing to promote healthy dietary behaviors supported the effectiveness of gain- vs. loss-framed messaging but few studies examined the effects of various dispositional factors [14,15,16,17].

Studies involving message framing to change dietary behaviors have primarily focused on messages that target behaviors of the person receiving the messages [14,15,16]. Few studies have investigated how message framing might be used to promote behaviors aimed at the health of someone other than the person receiving the messages (proxy) [18]. For example, parenting practices that affect the diet of children are completed by parents to directly benefit the health of their child. Having healthy children translates into benefits for parents such as limiting future healthcare costs and enhancing peace of mind [19], therefore message framing may produce similar results when the benefits directly impact the child and indirectly benefit the parent. Furthermore, few studies have examined the effectiveness of message framing to promote different behaviors that could achieve the same outcome [20]. For example, a variety of parenting practices can be used to promote the same healthy dietary behavior and health outcomes for children [20].

The purpose of this study was to test the hypothesis that exposure to gain-framed messages would result in greater intention (measured as motivation scores) for parents of children (6–12 years) for two different behaviors (role modeling intake and controlling home availability of SSBs) compared to exposure to loss-framed messages. Additionally, the effectiveness of gain- and loss-framed messages were tested among parents grouped by low and high scores on home availability of SSBs, low and high SSB intake, and normal, overweight, and obese weight status. 

## 2. Materials and Methods 

Parents/caregivers attending the Minnesota State Fair in 2015 were recruited to complete a questionnaire in a building specifically designed for research studies (Driven to Discover building) through a website providing study information. Parents were eligible if they had a child (6–12 years), had primary responsibility for food acquisition and preparation, and could complete the survey in English. The University Institutional Review Board approved the study with consent procedures. After participation, parents were given $5 in cash and a backpack.

Gain- and loss-framed messages were developed for parents promoting two parenting practices that addressed a single overall health outcome for children—role modeling intake and controlling home availability of beverages to limit SSB intake and improve diet quality and health (Table 1). Message phrasing was based on recent examples where gain-framed messages focused on benefits of engaging in the behavior and loss-framed messages focused on the costs of not engaging in the behavior [10,21,22]. For example, Churchill and Pavey [22] used the following gain- and loss-framed messages to promote fruit and vegetable intake, respectively: “Evidence suggests that people who eat enough fruit and vegetables, compared to those that do not, are at lower risk of many serious life-threatening diseases and gain several potential health benefits.” and “Evidence suggests that people who do not eat enough fruits and vegetables, compared to those that do, are at higher risk of many serious life-threatening diseases and lose several potential health benefits”.

An initial set of PowerPoint slides was developed presenting facts about the usual daily calories children consumed from added sugars (>10%), the relationship between high intake of sugary drinks and overweight or obesity, health risks associated with overweight or obesity, and prevalence of childhood obesity. For parents, sugary drinks were defined as sodas, fruit drinks, and fruit punch. These slides were followed by slides including the gain- and loss-framed messages for role modeling and controlling home beverage availability. The slides were transformed into short YouTube videos, tested with a small group of parents (*n* = 8) for clarity and comprehension, and revised accordingly prior to implementation with the large group.

For implementation, the revised slides were embedded as still images as part of a Qualtrics survey platform. The messages developed for each parenting practice are presented in Table 1. The order of the messages was randomized within the Qualtrics survey so different parents viewed the gain- vs. loss-framed messages in different order. Parents viewed the still images and then immediately reported behavioral intention (measured as motivation scores).

Questions to evaluate behavioral intention with respect to controlling availability of beverages and role modeling included: How much would this message motivate you to have healthy beverages at home for your child to drink (controlling home beverage availability)? and How much would this message motivate you to set a good example for your child by drinking healthy beverages (role modeling beverage intake)? Response options (motivation scores) were 1 = not at all—4 = a lot. These questions were similar to those used in another study evaluating motivation to engage in parenting practices based on messaging [23].

A subset of parents (*n* = 75) were asked if they perceived the messages to be framed according to the intended valence. Parents were asked whether the consequences of the parenting practices were described in a positive or negative way, which was a similar approach used in another study [24]. Response options were positive, negative, and I do not know. 

Home availability of beverages was measured with nine questions based on a similar questioning framework for foods available at home that had been used with parents of adolescents in a previous study [25]. The questions asked parents how often milk, soft drinks, fruit drinks, fruit juice, and water were available in their home with response options of never = 1—always = 4. Two items were grouped to construct a variable to assess the availability of sugar-sweetened beverages (regular soda pop and fruit drinks).

Usual beverage intake was assessed using a 15-item beverage questionnaire previously evaluated for validity and reliability as an indication of modeling beverage intakes [26]. Hedrick et al. [26] found that beverage intake measured with the 15-item beverage questionnaire was significantly correlated with intake measured with three 24-h dietary recalls (SSB R^2^ = 0.69), but not with whole milk. Various beverage items were included (dairy, sugar-sweetened, caffeinated, and energy beverages). Respondents were asked to indicate their usual intake over the past month by indicating how often they consumed the beverage (never or less than 1 per week, 1/week, 2–3/week, 4–6/week, 1/day 2+/day, 3+/day) and how much they consumed (less than 6 oz., 8 oz., 12 oz., more than 12 oz.). Items were grouped to construct variables to assess daily intake of SSBs including soft drinks, sweetened juice, sweetened tea, tea or coffee with cream and/or sugar, and energy drinks.

Parents answered questions assessing demographic and physical characteristics for themselves (age, sex, ethnicity, education, employment, food assistance, self-reported height and weight) and for their child (age, sex). Surveys were completed on iPads using a Qualtrics survey platform in about 10 min per participant. Parents first answered questions about home availability and usual beverage intake, followed by questions to evaluate behavioral intention based on viewing the gain- and loss-framed messages, and finally questions about demographic and physical characteristics.

For categorical variables, frequency counts were calculated. For continuous variables, means and standard deviations were computed. BMI (kg/m^2^) was calculated for parents from self-reported height and weight. Parents were divided into subgroups based on weight status (normal weight: BMI < 25, *n* = 130, overweight: BMI ≥ 25 and <30, *n* = 115, obese: BMI ≥ 30, *n* = 86); low and high home availability of SSBs based on summing ratings for two items, regular soda pop and fruit drinks, (below or above an availability rating of 5 (*n* = 170 and 210, respectively); and intake of SSBs (below or above median intake (*n* = 191 and 189, respectively). Paired *t*-tests were used to determine differences in behavioral intention (motivation scores) by type of message framing among all parents and within parent subgroups. Statistical significance was assessed at the *p* = 0.05 level. ANOVA models were used to assess between group differences in mean behavioral intention differences for each subgroup analysis. For the weight group comparisons, the Tukey adjustment for multiple comparisons was used to identify significant between group differences while preserving the overall alpha significance level at 0.05. Statistical Analysis System software (SAS; version 9.4, Cary, NC, USA) was used to analyze data. 

## 3. Results

Three-hundred and eighty parents completed the survey; an additional 7 parents provided consent but did not complete the survey. The majority were white (90.8%), women (79.7%), employed full time (72.9%), and had a 4-year college degree (70.5%); mean age was 42.0 years and mean BMI was 27.3 (Table 2). Twenty-eight percent of children were 6–8 years old, 72% were 9–12 years old and 50% were girls.

The manipulation check with 75 parents showed that a majority perceived the messages to be framed according to the intended valence. For the behavior of controlling availability of beverages for children, 85% of parents perceived the gain-framed message as positive; and 56% of parents perceived the loss-framed message as negative. For the behavior of role modeling beverage intake, 87% of parents perceived the gain-framed message as positive; and 76% of parents perceived the loss-framed message as negative.

A greater number of parents indicated that the gain-framed versus the loss-framed messages would motivate them (some and a lot) to control beverage availability at home (73.2% vs. 65.8%) and to role model beverage intake for their children (76.1% vs. 64.0%). Mean intention to control beverage availability in the home was greater (*p* = 0.002) after exposure to the gain-framed message (M = 3.06, SD = 0.90) compared to the loss-framed message (M = 2.93, SD = 0.96). Mean intention was also greater for role modeling beverage intake for children after exposure to the gain-framed message (M = 3.10, SD = 0.85) (*p* <0.001) compared to the loss-framed message (M = 2.81, SD = 1.03).

In the group with high availability of SSBs at home, exposure to the gain-framed message resulted in a higher mean intention to control availability of beverages at home (*p* < 0.010) compared to parents exposed to the loss-framed message (Table 3). However, in the group with low availability of SSBs at home, intention was not different after exposure to either the gain or loss framed message (*p* = 0.068). For parents consuming either a low or high amount of SSBs, intention to role model beverage intake was greater after exposure to the gain vs. the loss framed messages (*p* = 0.001) (Table 3). No differences were observed between low and high availability groups for intention to control availability of SSBs or between low and high SSB intake groups for intention to role model beverage intake (Table 3).

For both normal and overweight subgroups, no significant differences were observed in mean intention to control beverage availability at home after viewing the gain- and loss-framed messages (*p* = 0.066 and 0.668, respectively) (Table 4). For obese parents, the gain-framed message was more motivating than the loss-framed message regarding controlling availability. For all weight subgroups, gain-framed messages resulted in higher mean intention to role model beverage intake (*p* = 0.001) compared to loss-framed messages. No differences were observed in intention score differences to control availability of SSBs or role model SSB intake by weight subgroups.

## 4. Discussion

For all parents and within most parent subgroups, findings from the current study showed that gain-framed messages were related to greater motivation than loss-framed messages for both SSB parenting practices aimed at achieving the same outcome and focusing on behaviors that produced indirect benefits for the message recipient. Therefore the general principle that gain-framed messages are more effective than loss-framed messages in promoting prevention behaviors like healthy eating as shown in several reviews [10,18] is also likely to be applicable to messages targeting multiple parenting practices to improve diet and health of children.

Self-efficacy has been tested as a dispositional factor hypothesized to moderate the effectiveness of gain- or loss-framed health messages with inconclusive results [13]. Parent self-efficacy regarding SSB intake among youth has been addressed in previous studies [27,28,29]. Self-efficacy of parents was associated with SSB intake among young children in an observational study [27]. Self-efficacy was proposed as a control belief for serving SSBs to children by parents in a qualitative study [28]. Self-efficacy was also addressed through motivational interviewing as an intervention target to help parents control child SSB intake [29]. In the current study, the subgroup of parents with low home SSB availability may have been intentionally not keeping SSBs in the home to limit child intake based on strong self-efficacy for this parenting practice (although self-efficacy was not assessed). For these parents, no differences were observed in motivation based on gain- or loss-framed messages, thus the relationship between a potentially high level of confidence in limiting home availability of SSBs and a particular message valence remains unclear. Additional studies are needed to determine how self-efficacy affects motivation based on gain- or loss-framed messages promoting positive parenting practices. 

Level of involvement in a specific health issue is another dispositional factor that has been reviewed regarding effectiveness of gain- or loss-framed health messages [10,11,13]. For individuals with low involvement, gain-framed messages were generally more effective than loss-framed messages [10,13]. For the subgroup of parents in the current study with high availability of SSBs at home, involvement in controlling beverage availability for children may be low, consistent with the finding that gain-framed messages were more effective than loss-framed messages in improving motivation. 

In the current study, gain-framed compared to loss-framed messages resulted in greater motivation for role modeling beverage intake by all parents, and subgroups by SSB intake and weight status. However, parent beverage intake was assessed as an indication of role modeling and not measured with a general scaled variable. This approach was similar to that used in another recent study [30] and consistent with observed associations between parent and adolescent SSB intakes [31]. Therefore results based on subgroups by SSB intake may not be consistent with tests of the effectiveness of gain- vs. loss-framed messaging based on other measures of role modeling. 

Parental obesity has been associated with child obesity [32], increased likelihood that children and adolescents are less physically active [33], and a less supportive home food and physical activity environment [34]. Therefore, level of involvement in SSB parenting practices was expected to be low among the overweight/obese subgroup, indicating that gain-framed messages may have been more influential than loss-framed messages. This was the case for role modeling, where the gain-framed message was related to higher motivation scores versus the loss-framed message. However, no differences in effectiveness of message type were observed between normal and overweight parents for controlling availability. 

A strength of the current study was the novelty of testing effectiveness of gain- vs. loss-framed health messages to promote multiple behaviors that would benefit someone other than the message recipient. A limitation was that only a slight majority (56%) of parents perceived the loss-framed message as negative for controlling home beverage availability. Thus, caution should be used in interpreting the results regarding controlling home beverage availability. In addition, exposure to the messages was immediately followed by assessment of outcomes, whereas some parents may need time to process the information to influence behavior. Also, parents self-reported their height and weight, which could have led to over- or underestimation of the real values. Most parents were white, did not use food assistance programs and were well-educated women, thus limiting the ability to examine findings by ethnicity, food assistance, sex or education or to apply the findings to the broader population. 

In summary, given the positive findings regarding gain-framed messages and previous research on framing effects in nutrition education [10], future parent intervention programs may benefit from using gain-framed messages to promote parenting practices aimed at decreasing SSB intake by children. Further studies are needed to determine whether gain- compared to loss-framed messages are more effective in influencing actual change in SSB parenting practices and ultimately, child SSB intake and health outcomes.

## 5. Conclusions

Gain-framed messages promoting the SSB parenting practices of role modeling healthy beverage intake and making healthy beverages available were related to greater motivation scores than loss-framed messages for all parents. Within most parent subgroups based on low or high availability of SSBs, low or high SSB intake, and weight subgroups, gain- versus loss-framed messages were also related to higher motivation scores. Therefore gain-framed messages are recommended for parent interventions intended to decrease child intake of SSBs.

## Figures and Tables

**Table 1:** Gain- and Loss-Framed Messages.

Parenting Practice	Messages
Controlling Availability Gain-framed	Parents who do not make sugary drinks available in their home are more likely to have children who do not drink sugary drinks.
Controlling Availability Loss-framed	Parents who make sugary drinks available in their home are more likely to have children who drink sugary drinks.
Role Modeling Gain-framed	Parents who set a good example by not drinking sugary drinks are more likely to have children who do not drink sugary drinks.
Role Modeling Loss-framed	Parents who do not set a good example by drinking sugary drinks are more likely to have children who drink sugary drinks.

**Table 2:** Demographic Characteristics of Parent Survey Respondents.

Characteristic	Mean (SD)
Age (*n* = 380)	42.0 (6.6)
Body Mass Index (*n* = 331) ^1^	27.3 (6.0)
	N (%) ^1^
Sex	
Female	303 (79.7)
Male	77 (20.3)
Education	
High school diploma	21 (5.5)
Some college or technical school	91 (24.0)
4-year college, advanced degree	268 (70.5)
Ethnicity	
Hispanic or Latino	7 (1.8)
Asian	26 (6.8)
White/Caucasian	338 (88.9)
American Indian/Black/multi-ethnicity	9 (2.4)
Food Assistance Programs	
None	343 (90.3)
SNAP/WIC/Free or reduced price school meals) ^2^	50 (13.2)

^1^ BMI data are missing from 49; ^2^ SNAP—Supplemental Nutrition Assistance Program, WIC—Women’s, Infants and Children Supplemental Assistance Program. Participants could check all that apply.

**Table 3:** Behavioral Intention^1^ for Parenting Practices Based on Message Valence by SSB^2^ Availability and SSB Intake.

	Gain-Framed	Loss-Framed		Gain-Framed	Loss-Framed		
Parenting Practice	Mean (SD)	Mean (SD)	*p*-Value ^3^	Mean (SD)	Mean (SD)	*p*-Value ^3^	*p*-Value ^4^
	Low Availability SSB (*n* = 170)			High Availability SSB (*n* = 210)			
Controlling availability	3.01 (0.90)	2.90 (0.98)	0.068	3.12 (0.90)	2.98 (0.96)	0.010	0.296
	Low SSB Intake (*n* = 191)			High SSB Intake (*n* = 189)			
Role Modeling	3.21 (0.85)	2.86 (1.10)	0.0001	2.96 (0.83)	2.75 (0.95)	0.0005	0.125

^1^ Mean of response options 1–4, where 1 = not at all—4 = a lot; ^2^ SSB—sugar-sweetened beverages, ^3^ *p*-value based on paired *t*-test for differences in mean intention scores within groups, ^4^ *p*-value based on ANOVA F-test for between group differences in mean intention score differences.

**Table 4:** Behavioral Intention ^1^ for Parenting Practices Based on Message Valence by Weight Status.

	Normal Weight (*n* = 130)	Overweight (*n* = 115)	Obese (*n* = 86)	ANOVA *p*-Value ^3^
Controlling Availability	Mean (SD)	Mean (SD)	Mean (SD)	0.325
Gain-framed	3.19 (0.86)	3.03 (0.96)	3.05 (0.82)	
Loss-framed	3.06 (0.95)	2.99 (0.98)	2.84 (0.93)	
*t*-test *p*-Value ^2^	0.066	0.668	0.019	
Role Modeling	Mean (SD)	Mean (SD)	Mean (SD)	0.756
Gain-framed	3.17 (0.82)	3.19 (0.87)	3.06 (0.78)	
Loss-framed	2.93 (1.07)	2.85 (1.05)	2.72 (0.89)	
*t*-test *p*-Value ^2^	0.001	<0.0001	0.001	

^1^ Mean of response options 1–4, where 1 = not at all—4 = a lot; ^2^ *p*-value based on paired *t*-tests for differences in mean intention scores within weight subgroups, ^3^ *p*-value based on ANOVA F-test for between weight group differences in mean intention score differences.
